# Incidents in Porcelain Work

**Published:** 1907-04-15

**Authors:** J. M. Thompson


### INCIDENTS IN PORCELAIN WORK.

BY DR. J. M. THOMPSON.

Read before the Detroit Dental Society, January 10, 1907.

To write anything new upon this subject is difficult, and to present oft told tales is indeed irksome. However, with your kind indulgence I will try to conduct you through a few old paths, with perhaps a glimpse into a few which will be new.

After twenty years, porcelain as a filling material, has at last gained a permanent place in all up to date offices, and unless something better is produced, will stand at the head of the list for its tooth preserving as well as its artistic value.

Cranks (heritics as they are sometimes called) are necessary at the inception of any reform of great importance, and so it has been in the history of the use of porcelain in dental work. We, of Detroit have reason to feel a deeper interest in this work than dentists of other cities, on account of its being the home of the originator of the method now in use for måking inlays. It is not necessary at this time to enumerate the names of those who have been and are still the leaders in this work, but it must be a source of gratification to them to realize that so many are interested and working along with them.

This paper is to be given up to incidents in porcelain work and as incidents are usually the successes and failures of every day life a paper of this kind must necessarily relate the experiences of the writer, who now wishes to give full credit to those who have given out many valuable ideas through the journals,also to those of his associates in this society from whom he has received much that is good.

Operative Dentistry no longer stands upon the tripod of gold, amalgam and cement, but has four good legs to stand upon and while in the enthusiastic stage, some have told us that the use of porcelain is limited only by the skill of the operator, let us be thankful for the other materials and be skillful and judicious in their use.

Cavity preparation for inlays has been and is still a subject of much controversy. However, a few have grasped the real principles involved viz:—plenty of room for work, walls of cavity properly formed permitting an easy withdrawal of the matrix, at the same time forming a perfect seal for the inlay.

Time and space do not permit a discussion of the details pertaining to cavity preparation, but one of the incidents worthy of note is that shallow saucer-shaped cavities are a delusion and a snare, as are all cavities which lack clearly defined outlines and edges. Make all cavities as self retaining as possible and the cement will do the rest.

The forming of the matrix is the most important part of the work and will many times reveal fault in the cavity preparation which are easily overlooked. The angle from which we view a cavity does not always give us its true line of direction, and faulty edges are often shown in the platinum. In-as-much as the matrix is an exact reproduction of the cavity defects are plainly seen and easily corrected.

Several methods of forming the matrix have been taught and all have their advocates. The liberal mind selects the good from each and produces results which the man with only one idea can never accomplish. The man who believes the impression method to be the only one, limits his field of usefulness as truly as does the man who stakes his reputation upon his ability to burnish foil into any and every place.

Labial and buccal cavities are reduced to simple tasks by the combination of these two methods and the results (to say nothing of the time saved) are very gratifying. Let us take for example a labial cavity in an upper incisor. Supposing we have not the time to press the gum away from the cervical border and an inlay is immediately required. Here the impression man would have the burnishing man at a sad disad vantage. Why? Because it is next to impossible to work thin platinum up under the gum and keep it from slipping away from the margin and not be drawn in the cavity, giving no defined line of the cervical border whatever. On the other hand with a piece of dental lack an impression can be made in five minutes and in five more a swaged matrix can be made.

The technique of taking an impression of a labial cavity is as follows:—A piece of dental lack about the size of a buckshot is warmed over an alcohol flame and with the fingers forced into the cavity and the surplus flatened against the tooth and gum. Upon cooling, it is then removed and placed upon the ring of an Ajax swager which is already full of the same material. By warming the surface of the lack in the ring the impression may be firmly seated in it without any change of form. A piece of platinum is now cut to fit and carefully burnished to conform in a general way to the outline of the impression. This is absolutely necessary as the platinum will tear unless it is done. When this has been accomplished a disk of rubber dam is laid over the platinum and the ring placed in the swager. By using gentle blows at first and then driving to place an accurate reproduction of the impression is produced. The disk of rubber dam prevents the platinum from sticking to the soft rubber used in the swager and saves the distortion which would necessarily follow without its use. After its removal from the impression the matrix may be trimmed and adjusted to the cavity for the burnishing. It may be ligated into place and burnished to a perfect fit, then packed with camphor gum and removed.

Whoever gave the use of camphor to inlay workers deserves much credit as it has three very valuable qualities, viz., it is a perfect swager, has rigidity enough to preserve form of the matrix during its removal from the cavity and it burns out without a trace of its having deen used.

Many other cavities may be treated in the same manner and while the best results are obtained from the matrix burnished directly to the cavity , the impression gives us a prepared form which enables us to force the platinum into all parts of the cavity without much danger of rocking or tearing.

We now come to the placing of the porcelain in the matrix . Here is where good judgment is needed most of all. Warpage caused by the shrinkage of the porcelain is one of the great points of consideration. Several methods of overcoming this difficulty are taught, but the two possessing the greatest value are first the coating of the matrix with the shellac which permits the porcelain to draw away from the sides, thereby leaving a space to be filled in , and secondly by filling the matrix to its desired fullness and with a very thin knife splitting it through the center. This permits the shrinkage to take place from the center and the edges are covered and protected by a good layer of body.

An inlay made in this way may be returned to the cavity and its contour noted without danger of harm to the edges. An instrument may be placed between the two halves and the matrix forced against the walls of the cavity. It is then removed and again filled to the desired contour, two or three backings are all that are necessary. These methods are used principally in molar and bicuspid inlays.

In placing the foundation body in the matrix prepared for the contour of an incisor, as much as is needed should be placed in at once, and if the filling be a large one it is always best to split it about half way between the cervical border and the cutting edge. When using porcelains which have different fusing points it is best to fill in the opening left by the shrinkage with the material first used and then add the enamel bodies. In using low fusing material upon a higher fusing foundation never fill the matrix full but add small amounts of each color as needed always keeping the outer margins of the matrix free from any accumulations of enamel until enough has been baked to entirely finish the work.

We now have on the market for inlay purposes—Jenkins' & Brewster's low fusing, Brewster's so called high fusing, S. S. Whites, Whiteleys and the Consolidated. Any one thoroughly conversant with the fusing of the different kinds can take any of them and do good work. Now this may seem a very liberal statement, but I know whereof I make it. There is more of a field for low fusing bodies in inlay work than there is in crown and bridge work, and in continuous gum work, the low fusing body has no place at all.

The shadow problem in inlay work is one that is to a certain extent hard to solve. Mr. Brewster thought to overcome this by the use of a pure white foundation body which would radiate sufficient light and also prevent the cement from producing a change of color. In large fillings, especially those for labial cavities the cement has a great deal to do with the appearance and these call for an opaque rather then a translucent porcelain. Jenkin's or Brewster's materials are indicated here more than any others.

The difficulty in using the pure white furnished by Mr. Brewster in shallow labial cavities is that if by accident it is fused to a high glaze, it has such a luster that it is hard to overcome it with the colors necessary in the little space remaining.

In contours , high fusing bodies give the best satisfaction. The work of building is done in much less time than with the low and they have many advantages. A fine edge is obtained, an inlay can be made in three fusings, and if when the platinum is removed it is found lacking in contour, Brewster's enamel may be added and fused without any fear of injury to the edges while this is being done.

Moist porcelain is readily dried by touching it to a little of the dry powder; by gently touching with a brush that which adheres to the surface of the part we are drying, it breaks away leaving a smooth surface.

*Fusing.* Dr. Byram in his paper read in Chicago last year told us porcelain has no fixed fusing point. While he was one of the first of recent writers to call marked attention to the fact many others had been forced to believe it by finding their work ruined by carelessly leaving it in the furnace which was running at its lowest temperature.

Better results are obtained by giving a longer time at a low temperature than by a high temperature and a short time.

A pyrometer will show you what a difference of 50 or 100 degrees will do to your work at the time when a slight over bake would ruin it.

There never has been and there never will be an automatic device made which can be trusted at all times.

There are several reasons why this is so.

First of all is the fact which we have just mentioned, i. e. that porcelain has no fixed fusing point.

Secondly, that no matter from what source current is obtained it will vary from ten to fifteen volts, giving us a high and a low pressure.

Thirdly, a new muffle will always fuse faster than an old one.

Fourthly, the difference in the size of the slab upon which work is carried into the muffle will make a difference in the time of fusing.

Fifthly, drafts of air will sufficiently cool a furnace so as to show a deflection of the indicator from 50 to 100 degrees.

To refer to our third point mentioned (the new muffle and the old one). Those who have used an electric furnace and are of an inquiring mind, have found that after a muffle has given out that the platinum wire is very brittle and in places will be encrusted with a silicate formed from the material of which the muffle is made. The wire gradually diminishes in size, consequently has not the carrying apacity to generate heat which it had in the beginning, and as this process takes place during the continued use of the furnace it will take a longer time to reach a given temperature until such a time as it gives out entirely.

This goes to show that working without a pyrometer one is to a great extent groping in the dark. It also proves that we cannot depend upon the pyrometer alone and that it must be timed, although it is not necessary to watch it as closely as when working without it. The pyrometer, however, as an aid in the working of porcelain, is almost indispensable, and its greatest benefit is its relieving one of the necessity of looking into the furnace, or ia any way giving the eyes the severe test which they must necessarily be given when depending upon them to determine the progress of our work.

To refer to our fourth point (the difference in the size of the slab upon which the work is carried to the muffle) only a few appreciate what a difference in time this may cause. For instance the normal temperature of a muffle is generally 1800 degrees. When one of the large slabs, such as are furnished with the Pelton furnace, is placed in the muffle it will reduce the temperature from 300 to 400 degrees, and the small round slab will reduce about half as much. This does not make any great difference, but it only goes to show that our use of the appliances must be uniform to secure uniform results. The position which the work occupies in the muffle also has something to do with the general results, and too much attention to these details is impossible. Work placed upon too thick a slab will be underdone at the base and over-done at the top.

A few points in connection with the use of cements—Cement which is to be used for setting inlays in conspicuous places should never be mixed with a metal Spatula, a ground glass slab and an orange wood Spatula give the best results.

In setting inlays in approximal cavities a strip of rubber dam drawn over the cavity adjoining prevents the smearing of its edges with the cement prepared for the other.

When an inlay is placed in a cavity, it should be forced to place and pressure for retention applied immediately and not increased later. Trying to make a closer fit after the cement has commenced to set, results in chipped edges and unsightly work.

A celluloid matrix is also indicated for these cavities as it protects one and at the same time makes an excellent compress and guide for the inlay after it is placed in the cavity.

### DISCUSSION.

DR. C. H. LAND: I am grateful to this society for the opportunity you extend to me for discussing the subject of porcelain inlays before you, and this able presentation of Dr. Thompson. I have been called an enthusiast and a crank on this subject in the days that are gone; but I have tried to have the satisfaction of knowing that it is today an honor to have labored in the cause of the porcelain inlay. I applied, unsuccessfully, twenty-two years ago for a patent on porcelain and gold inlays, and in 1887 I contributed an article to the Independent Practitioner giving my process to the profession. I thought I had made a real discovery and organized a company to carry out my ideas, and lost fifteen thousand dollars before I got through that experiment. In 1887 I reported 136 cases, in practice at that time, to the Michigan State Dental Association.

I believe the present day workers are making a mistake in trying to use porcelain bodies that fuse at too low temperatures. The electric furnaces are not capable of fusing bodies that should be used to get ideal results. The platinum wire soon disintegrates under high temperatures required to fuse good porcelain. For this reason I invented my gas and oil furnaces. I can fuse porcelain that would burn out any of the present electric muffles. The porcelain used at present contain to much flux. This weakens the porcelain and causes it to craze or cheek if not carefully annealed, in fact porcelain for inlay purposes should contain no flux. True porcelain may be fired under bad conditions and it will never cheek. If we should use a high grade porcelain and could carve out teeth or build them up with it, we should get more artistic results.

When the high grade porcelains are used the colors are more substantial. I do not believe in using more than one grade of porcelain in the same tooth, it creates a tendency to craze or cheek. A porcelain made of 75 parts Kaolin and 40 parts Feldspar to which a little Silica is added will be translucent and produce satisfactory results. The present method of fusing with a pyrometer and an electric furnace will not give uniform results, because you have no chance to circulate oxygen or air through the muffle while baking, consequently your colors will not fuse out perfectly. The so called low fusing porcelains are not much better than so much glass and should never be used when resistance to mechanical or chemical stress is great. It is important that the temperature of the furnace should be uniform, and no chances for sudden or excessive changes should be tolerated, else you may destroy the color, or check the inlay, or warp the matrix. A large muffle slab is desirable as it will hold the heat so as to make the cooling process slower and more satisfactory.

Dr. E. B. Spalding: If it has taken Dr. Land nearly thirty years to learn what he knows about porcelain, some of us will be getting discouraged; but the fact is we have had a lot of the experimenting done for us by the pioneers, and we can make good use of their inventions. The essayist says that shallow inlays are a snare and delusion. If we make our labial inlays as deep as is practicable with definite margins nearly parallel walls with angular floor angles they will set firmly and securely. A shallow and indefinitely shaped cavity will not retain an inlay however good the cement, as the angular form determines the placement so definitely that the cementing process is made easy and certain.

It is not desirable to make large extension on incisal or morsal surfaces, and where attenuated margins are made, the inlay had better be made of gold. I burnish practically all my inlays, the platinum can be drawn from the center of the cavity to the margin or from the tooth surface to the margin, so that little force need be used against the sharp marginal angles. I don't believe much in the idea of cementing under continued pressure as one is so liable to disturb the cement while it is crystalizing. Force the inlay to its position and don't disturb it until the cement has thoroughly set. I have used with gratifying results a mixture of Ames brown and gray cement in the proportion of one tenth to one fifth of the brown cement; the tone result is much more satisfactory than when the single light color is used.

Dr. George Burke: I once heard Dr. Land say that where porcelain inlays were most needed they were most likely to fail and I have frequently found this to be so. I am of the opinion that we have lost sight of mechanical forms of retention and depended too much on the cement for retention only to have a failure to report. I am convinced that we shall not soon get away from the inlay method of filling teeth although I do not make as much use of it as I should, I presume like most men who have been educated to work the metallic filling material, we find it difficult to adopt a principle which is antogonistic in much of its technique. But as time goes on and we meet with better success I believe we shall get sufficient confidence to assure our faltering indecision.

Dr. Land: A contour filling differs from a true inlay, and should always be enforced by some mechanical means of retention. A post or pin is generally used, I have increased the retention form by fusing fine silica to the cavity surface of an inlay. Oxide of zinc fused to cavity surface of inlay will also increase cement attachment. The gold inlay will not hold the cement as well as the etched porcelain, and in my opinion the gold inlay has no advantage over a gold filling, and in most cases a porcelain inlay will serve as good a purpose.

